# An exhaustive inventory of coniferous trees in an agricultural landscape

**DOI:** 10.3897/BDJ.3.e4660

**Published:** 2015-02-23

**Authors:** Jérôme Rousselet, Alain Roques, Jacques Garcia, Jean-Pierre Rossi

**Affiliations:** ‡ INRA, UR633 Zoologie Forestière, Orléans, France; § UMR CBGP (INRA/IRD/Cirad/Montpellier SupAgro), Montferrier-sur-Lez, France

**Keywords:** Ornamental trees, pine processionary moth, point pattern, trees outside forests, open-field landscape

## Abstract

**Background:**

Various species of forest trees are commonly used for ornamental purposes and are therefore frequently found in non-forest ecosystems. These trees constitute a significant component of the trees outside forests (TOF). Although increasingly recognized as prominent feature of agricultural lands and built-up areas, not much is known, however, about TOF since they are generally absent from forest inventories.

**New information:**

In the present study, we focus on the coniferous tree species that constitute potential hosts for a forest defoliator, the pine processionary moth *Thaumetopoea pityocampa* Den. & Schiff. (Lepidoptera, Notodontidae). We carried out an exhaustive inventory of all pines (*Pinus* spp.), cedars (*Cedrus* spp.) and Douglas-fir (*Pseudotsuga menziesii*) in a 22 × 22 km study window located in the open-field region of Beauce in the centre of France. We recorded a total of 3834 individuals or small groups host trees corresponding a density of 7.9 occurrences per 100 ha. We provide the spatial coordinates of the points without differentiation between tree species.

## Introduction

Forest trees are commonly used for ornamental purposes and are therefore frequently found in non-forest ecosystems, especially in urbanized areas. These trees constitute an important component of the so-called trees outside forests (TOF) defined as “trees on land not defined as forest and other wooded land”, a definition that is dependent on how forests and woodlands are defined themselves (Kleinn 2000). TOF provide various ecosystem services, e.g., control over soil erosion, nutrient and water cycling, biodiversity conservation or pest control, but despite a growing interest (de Foresta et al. 2013), data documenting TOF are still scarce especially in large open-field agroecosystems. A substantial proportion of TOF are ornamental or amenity trees grown for decorative purposes in gardens and landscape design projects.

**Figure 1.**
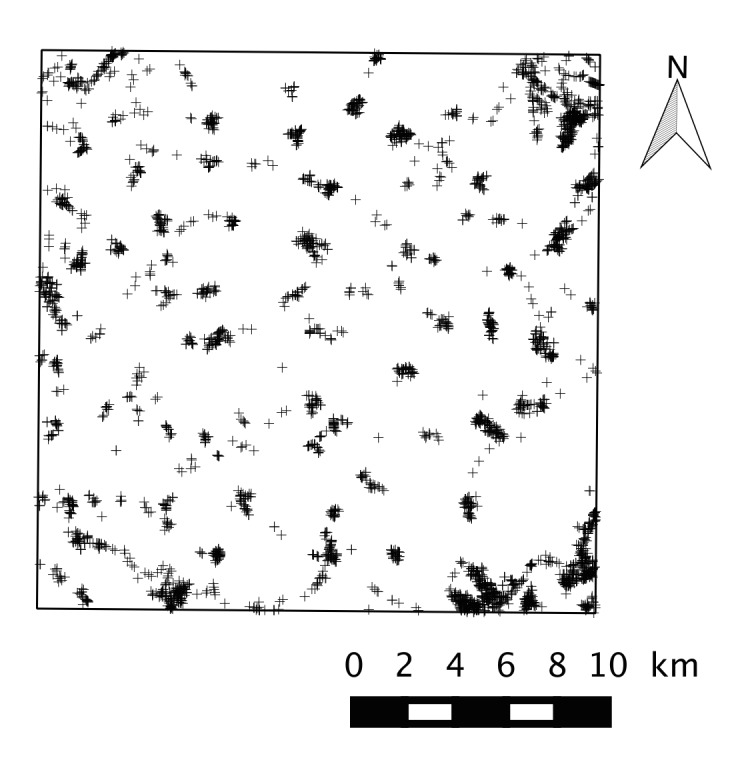
Survey plot (22 × 22 km) where an exhaustive inventory of all trees belonging to the genera *Pinus*, *Cedrus* and *Pseudotsuga* was carried during autumn and winter 2009–2010. Trees are indicated by crosses.

In the present study, we focus on the coniferous tree species that are potential hosts for a forest defoliator, the pine processionary moth *Thaumetopoea pityocampa* Den. & Schiff. (Lepidoptera, Notodontidae) (thereafter referred to as PPM). *T. pityocampa* feeds on pines (*Pinus* spp.), cedars (*Cedrus* spp.), and occasionally on Douglas-fir (*Pseudotsuga menziesii*) (Cielsa 2011, Roques 2015). The aim of the present survey was to carry out an exhaustive inventory of TOF constituting suitable hosts for the PPM in a 22 × 22 km study window located in the open-field region of Beauce in the centre of France (Fig. 1). Single trees, linear groups of trees and small woodlands were identified and geolocalized, and the resulting data set can be used to assess tree spatial pattern or their contribution to important landscape features (e.g. connectivity) with regards to forest organisms such as the PPM.

The geographical range of the PPM is currently expanding northward in relation to climate change (Roques 2015). During this process, the moth colonizes various non-forest areas where TOF are likely to play a key role by facilitating its dispersal especially in large open-field regions. The data reported here were specifically collected to assess how TOF that are suitable for hosts for the PPM contribute to landscape connectivity for that specie. For that reason, we did not record the tree species identity but only their status as hosts (or not). We used the data set for landscape analyses (Rossi et al. under review) and point process analyses (Rossi and Rousselet 2015). Since tree species were not recorded, we provide here the points locations without any taxonomic taxonomic information. Since - to our knowledge - there is no comparable inventory available in the literature, we publish the data set with the hope that it is useful to other researches such as comparative and/or new data analyses.

## Sampling methods

### Study extent

Because the study was carried out in metropolitan France, we used the official projection RFG93 Lambert-93 (Réseau Géodésique Français 1993 - EPSG code 2154; http://spatialreference.org/ref/epsg/rgf93-lambert-93/). The bounding box of the survey plot is given in Table 1.

### Sampling description

An exhaustive inventory of PPM host trees outside forests was carried out in a 22 × 22 km = 484 km^2^ area located in the north of the Centre region of France (Fig. 1). All trees belonging to the genera *Pinus*, *Cedrus* and *Pseudotsuga* were considered as potential hosts. The study site was located in the southern part of an ecoregion referred to as the Drouais-Thymerais region (Conseil régional Centre 2009) where the landscape mostly consisted of wide arable lands (cereal). All the roads and all the tracks suitable for cars were visited during autumn and winter 2009–2010. This period of the year was preferred because sighting and identification evergreen coniferous species is easier in winter when deciduous trees have lost their leaves (Rousselet et al. 2013). Every individual or small group of host trees (single trees, linear groups of trees, small woodlands) was observed by eye, and with binoculars when necessary, from the road and public land. The geographic coordinates were recorded using a Garmin ^TM^ GPS12. The vendor specifies a RMS accuracy of the coordinates to be 15 meters.

## Geographic coverage

### Description

France

### Coordinates

South: 48.467738 decimal degrees and North: 48.667878 decimal degrees Latitude; West: 1.538854 decimal degrees and East: 1.237294 decimal degrees Longitude.

## Taxonomic coverage

### Description

The inventory accounted for all tree species potentially hosting the PPM. In the study area, this corresponded to the genera *Pinus*, *Cedrus* and *Pseudotsuga*. Species were not distinguished when trees spatial location were recorded.

## Temporal coverage

### Notes

Data were collected between autumn 2009 and winter 2010.

## Usage rights

### Use license

Creative Commons CCZero

## Data resources

### Data package title

Coniferous trees inventory in the Beauce region

### Number of data sets

1

### Data set 1.

#### Data set name

Coniferous trees in a 22 by 22 km plot in Beauce

#### Data format

text in csv format

#### Number of columns

4

#### Description

**Data set 1.** 

Column label	Column description
longitude_EPSG2154	Trees longitude in the coordinate reference system EPSG code 2154 (RFG93)
latitude_EPSG2154	Trees latitude in the coordinate reference system EPSG code 2154 (RFG93)
longitude_EPSG4326	Trees longitude in the coordinate reference system EPSG code 4326 (WSG84)
latitude_EPSG4326	Trees latitude in the coordinate reference system EPSG code 4326 (WSG84)

## Supplementary Material

Supplementary material 1 An exhaustive inventory of coniferous trees in an agricultural landscape Data type: spatial coordinates
Brief description: The file contains the spatial coordinates of the trees that are potential hosts for the pine processionary moth *Thaumetopoea pityocampa* Den. & Schiff. (Lepidoptera, Notodontidae) in a 22 × 22 km study window located in the open-field region of Beauce in the centre of France. Considered trees are pines (*Pinus* spp.), cedars (*Cedrus* spp.) and Douglas-fir (*Pseudotsuga menziesii*).
File: oo_37992.xml Jérôme Rousselet, Jacques Garcia, Alain Roques and Jean-Pierre Rossi

Supplementary material 2 An exhaustive inventory of coniferous trees in an agricultural landscape Data type: spatial coordinates in xls format
Brief description: The file contains the spatial coordinates of the trees that are potential hosts for the pine processionary moth Thaumetopoea pityocampa Den. & Schiff. (Lepidoptera, Notodontidae) in a 22 × 22 km study window located in the open-field region of Beauce in the centre of France. Considered trees are pines (Pinus spp.), ccedars (Cedrus spp.) and Douglas-fir (Pseudotsuga menziesii).
File: oo_38110.xls Jérôme Rousselet, Jacques Garcia, Alain Roques and Jean-Pierre Rossi

## Figures and Tables

**Table 1.** Study plot bounding box

	Longitude (EPSG 2154)	Latitude (EPSG 2154)	Longitude (EPSG 4326)	Latitude (EPSG 4326)
bottom left	570017.01063056453	6820055.4608164523	1.241456	48.467738
bottom right	592001.36005055474	6819872.1581092915	1.538854	48.470121
top right	592184.85387388722	6841856.7000891836	1.535822	48.667878
top left	570200.12007769709	6842040.0006731432	1.237294	48.665486
